# Molecular Signature of Endometrial Cancer with Coexistent Adenomyosis: A Multicentric Exploratory Analysis

**DOI:** 10.3390/cancers15215208

**Published:** 2023-10-30

**Authors:** Diego Raimondo, Antonio Raffone, Agnese Virgilio, Stefano Ferla, Manuela Maletta, Daniele Neola, Antonio Travaglino, Roberto Paradisi, Alicia Hernández, Emanuela Spagnolo, Virginia García-Pineda, Jacopo Lenzi, Maurizio Guida, Paolo Casadio, Renato Seracchioli

**Affiliations:** 1 Division of Gynecology and Human Reproduction Physiopathology, IRCCS Azienda Ospedaliero-Universitaria di Bologna, 40138 Bologna, Italy; 2 Department of Medical and Surgical Sciences (DIMEC), University of Bologna, 40138 Bologna, Italy; 3 Gynecology and Obstetrics Unit, Department of Neuroscience, Reproductive Sciences and Dentistry, School of Medicine, University of Naples Federico II, 80138 Naples, Italy; 4 Unit of Pathology, Department of Medicine and Technological Innovation, University of Insubria, 21100 Varese, Italy; 5 Department of Gynecology, La Paz University Hospital, Hospital Universitario La Paz, Paseo de la Castellana, 261, 28046 Madrid, Spain; 6 Department of Gynecologic Oncology, La Paz University Hospital, Hospital Universitario La Paz, Paseo de la Castellana, 261, 28046 Madrid, Spain; 7 Department of Biomedical and Neuromotor Sciences, University of Bologna, 40126 Bologna, Italy

**Keywords:** endometrial carcinoma, molecular pattern, tumor, prognosis, neoplasm, malignancy, immunohistochemistry, endometriosis, endometrium, genome

## Abstract

**Simple Summary:**

The impact of the molecular signature on the favorable prognosis of endometrial cancer patients with coexistent adenomyosis is undefined. We aimed to compare the prevalence of molecular groups at poor and intermediate prognosis (p53-abn and MMR-d groups) between endometrial cancer patients with and without coexistent adenomyosis through a multicentric, observational, retrospective, cohort study. We included 147 endometrial cancer patients (38 in the adenomyosis group and 109 in the no adenomyosis group) and we found no significant difference in the prevalence of p53-abn (*p* = 1.000) and MMR-d (*p* = 0.2880) signatures between the two groups. Therefore, the molecular signature does not appear to explain the better prognosis associated with coexistent adenomyosis in endometrial cancer patients. Further investigation of the topic requires future larger studies.

**Abstract:**

Adenomyosis has been associated with better survival outcomes in women with endometrial cancer. However, although the endometrial cancer patients’ risk stratification has been revolutionized by molecular findings, the impact of the molecular signature on the favorable prognosis of endometrial cancer patients with coexistent adenomyosis is unknown. The aim of our study was to compare the prevalence of molecular groups at poor and intermediate prognosis between endometrial cancer patients with and without coexistent adenomyosis. A multicentric, observational, retrospective, cohort study was performed to assess the differences in the prevalence of p53-abnormal expression (p53-abn) and mismatch repair protein-deficient expression (MMR-d) signatures between endometrial cancer patients with and without coexistent adenomyosis. A total of 147 endometrial cancer patients were included in the study: 38 in the adenomyosis group and 109 in the no adenomyosis group. A total of 37 patients showed the MMR-d signature (12 in the adenomyosis group and 25 in the no adenomyosis group), while 12 showed the p53-abn signature (3 in the adenomyosis group and 9 in the no adenomyosis group). No significant difference was found in the prevalence of p53-abn (*p* = 1.000) and MMR-d (*p* = 0.2880) signatures between endometrial cancer patients with and without coexistent adenomyosis. In conclusion, the molecular signature does not appear to explain the better prognosis associated with coexistent adenomyosis in endometrial cancer patients. Further investigation of these findings is necessary through future larger studies.

## 1. Introduction

Endometrial cancer is the most frequent gynecological neoplasm in Western countries [1,2,3,4,5,6,7,8]. The risk stratification of endometrial cancer patients had been mainly based on post-surgical pathological prognostic factors prior to the introduction of the Cancer Genome Atlas (TCGA) Research Network molecular classification [9] and the Proactive Molecular Risk Classifier for Endometrial Cancer (ProMisE) [1,2,4,10,11]. According to the ProMisE, endometrial cancer patients are classified into four molecular groups with different prognosis [1,2,4,10,11]: ultramutated group, characterized by mutations in the exonuclease domain of Polymerase-ε (POLEmt) and good prognosis; hypermutated group, characterized by mismatch repair (MMR) protein-deficient immunohistochemical (IHC) expression (MMR-d) and intermediate prognosis; copy-number high group, characterized by abnormal p53 IHC expression (p53-abn) and the worst prognosis; and copy-number low group, characterized by good to intermediate prognosis and lack of specific molecular profile (NSMP) [1,2,4,10,11]. Nowadays, the integration of ProMisE groups with conventional pathological prognostic factors is recommended by the international guidelines [1,2,4,10,11] in order to further tailor the risk stratification and adjuvant treatment of endometrial cancer patients.

Adenomyosis is a disease defined by the presence of ectopic endometrium within the myometrium [12,13]. It has been reported in up to one in four patients with EC [12], representing a factor associated with better survival outcomes [14]. Several hypotheses have been made to explain such a difference in prognosis between endometrial cancer patients with and without coexistent adenomyosis. In particular, differences in the prevalence of pathological, clinical and/or molecular prognostic factors between women with and without coexistent adenomyosis have been supposed. In detail, while clinical factors (with the exception for nulliparity) have shown no significant differences between endometrial cancer patients with and without adenomyosis, conventional pathological prognostic factors have been shown to be less frequently unfavorable in endometrial cancer patients with adenomyosis compared to endometrial cancer patients without adenomyosis [8,14]. However, to our knowledge, data about molecular prognostic factors (i.e., molecular groups) in endometrial cancer patients with coexistent adenomyosis have never been reported; thus, the impact of the molecular signature on the reported favorable prognosis of endometrial cancer patients with coexistent adenomyosis is unknown.

The aim of this study was to compare the prevalence of molecular groups at poor and intermediate prognosis (i.e., p53-abn and MMR-d groups) between endometrial cancer patients with and without coexistent adenomyosis.

## 2. Materials and Methods

### 2.1. Study Protocol

Following an a priori defined study protocol, this study was designed as a multicentric, observational, retrospective, cohort study.

The STrengthening the Reporting of Observational Studies in Epidemiology (STROBE) statement and checklist [15] were used to report this study.

### 2.2. Selection Criteria

Medical records and electronic clinical databases were consulted for all consecutive patients who underwent surgical staging for endometrial cancer and IHC evaluation for MMR protein and p53 expression on final surgical specimens, at two tertiary level referral centers for gynecological cancers (S. Orsola Hospital, University of Bologna, Bologna, Italy; and Department of Gynecologic Oncology, La Paz University Hospital, Hospital Universitario La Paz, Madrid, Spain) from September 2020 to September 2022. The exclusion criteria were the absence of IHC assessment on final surgical specimens and incomplete surgical staging.

The endometrial cancer patients were divided into two groups based on the presence of adenomyosis on final surgical specimens, assessed by experienced gynecological pathologists: patients with coexistent adenomyosis (adenomyosis group) and patients without coexistent adenomyosis (no adenomyosis group). Adenomyosis was diagnosed based on the presence of ectopic endometrium (seen at least 2.5 mm or more than one microscopic field at 10× magnification from the endomyometrial junction), circumferentially surrounded by a bundle of hypertrophic smooth muscle cells [16,17].

### 2.3. Study Endpoints

The study endpoints were the differences in the prevalence of molecular signatures at poor and intermediate prognosis (i.e., p53-abn and MMR-d) between endometrial cancer patients of the adenomyosis group and those of the no adenomyosis group.

Immunohistochemical analysis was performed as per routine clinical practice. In fact, IHC was found as a highly accurate surrogate of gene sequencing in detecting TP53 mutations and microsatellite instability [18,19]. In particular, MMR protein IHC expression was categorized as “proficient” (MMR-p) in the case of positive nuclear expression of 2 MMR proteins (MSH6 and PMS2), and “deficient” (MMR-d) in the case of loss of nuclear expression of the MSH6 and PMS2 proteins in at least 10% of the tumoral area, in the presence of an internal positive control (stromal cells and lymphocytes) [19]. Regarding p53 IHC expression, it was categorized as “normal” (p53-wt) in the case of nuclear expression in <70–80% of tumor cells with heterogeneous intensity, and “abnormal” (p53-abn) in the case of (i) strong nuclear expression in ≥70–80% of tumor cells (with or without cytoplasmic expression) and (ii) complete loss of nuclear expression in the presence of an internal positive control (stromal cells and lymphocytes) [19]. According to ProMisE [18], endometrial cancers with both MMR-d and p53-abn signatures were classified as MMR-d cases. On the other hand, endometrial cancer patients with either the MMR-d or p53-abn signature were screened for the presence of mutations in the exonuclease domain of POLE through sequencing [18,20]; in fact, cases with both MMR-d and POLE-mt, and cases with both p53-mt and POLE-mt signatures were considered as POLE-mt and, therefore, not included in our study population. Indeed, as our centers restrict POLE testing to only those patients in whom this would alter adjuvant therapy recommendations in order to restrict sequencing costs, the POLE signature was not available for all endometrial cancer patients. Thus, a retrospective calculation of the prevalence of the POLE signature (and subsequently of the NSMP signature) was not feasible. Based on these considerations, during the elaboration of the study protocol, we a priori decided to only assess p53-abn and MMR-d signatures.

Other collected data for each included patient were the following: age at diagnosis, body mass index (BMI), tumor histotype and grade, depth of myometrial invasion, lymphovascular space invasion (LVSI), International Federation of Gynecology and Obstetrics (FIGO) staging, lymph node involvement, adjuvant treatment, disease recurrence, and any deaths during follow-up for causes independent of or dependent on endometrial cancer.

### 2.4. Statistical Analysis

Continuous data were described as mean ± standard deviation (SD) or median (interquartile range), as appropriate. Categorical variables were presented as numbers and percentages. Student’s *t*-test or Mann–Whitney U-test and χ^2^ or Fisher’s exact test were used for continuous and categorical data, respectively, as appropriate. A value of *p* < 0.05 was considered significant for all tests. Statistical analysis was carried out using the SPSS software version 24.0 (IBM Corp., Armonk, NY, USA).

#### Post hoc Analysis

Based on the sample size of 38 and 109 patients with and without adenomyosis and the observed prevalence of the MMR-d signature of about 32% and 23% in the two groups, respectively, which gives an odds ratio of 1.55, we obtained a retrospective power for a two-sample proportions test of only 21%.

### 2.5. Ethical Statement

The Institutional Review Board of the IRCCS Azienda Ospedaliero-Universitaria di Bologna (CE-AVEC 389/2021/Oss/AOUBo) approved this study which was conducted in accordance with the Helsinki Declaration. An informed consent form was signed by all patients for the use of their anonymized data for research purposes.

## 3. Results

During the study period, 220 women underwent surgical staging for endometrial cancer. Seventy-three patients were excluded since they did not meet the selection criteria. Finally, 147 endometrial cancer patients were included in the study: 38 (25.8%) in the adenomyosis group and 109 (74.1%) in the no adenomyosis group.

Median age (range) at surgery was 62 (38–84) years in the adenomyosis group and 64 (28–92) years in the no adenomyosis group. Most of the endometrial cancer showed an early FIGO stage (89.5% in the adenomyosis group and 82.6% in the no adenomyosis group), low grade (78.9% in both groups) and endometrioid histotype (89.5% in the adenomyosis group and 84.4% in the no adenomyosis group).

No statistically significant difference between adenomyosis and no adenomyosis group was found in terms of age, BMI, FIGO stage, histotype, tumor grade, LVSI, sentinel lymph node involvement and adjuvant treatment.

Regarding survival outcomes, five (3.4%) recurrences (two in the adenomyosis group and three in the no adenomyosis group) were observed during the follow-up (median follow-up time: 18 months) (Table 1). In particular, in the no adenomyosis group, two patients with advanced FIGO stage and high-grade endometrioid endometrial cancer experienced recurrence after 6 and 17 months, while another patient with low-grade early-stage endometrioid endometrial cancer showed recurrence 18 months after surgery. On the other hand, in the adenomyosis group, two recurrences occurred at 12 months and 24 months after surgery for early-stage low-grade endometrial cancer. Moreover, one death due to endometrial cancer was reported in the adenomyosis group: a 61-year-old woman developed local recurrence and distant metastasis and died 24 months after surgery and brachytherapy for IB FIGO stage, low-grade endometrioid endometrial cancer which showed the MMR-d signature.

Concerning the study endpoints, 37 (25.2%) patients showed the MMR-d signature (12 in the adenomyosis group and 25 in the no adenomyosis group), while 12 (8.2%) showed the p53-abn signature (3 in the adenomyosis group and 9 in the no adenomyosis group). No significant difference was found in the prevalence of p53-abn (*p* = 1.000) and MMR-d (*p* = 0.2880) signatures between endometrial cancer patients with and without coexistent adenomyosis (Table 2).

## 4. Discussion

### 4.1. Main Findings and Interpretations

This study showed no significant difference in the prevalence of p53-abn and MMR-d signatures between endometrial cancer patients with and without coexistent adenomyosis. This would indicate that the molecular signature is not among the prognostic factors underlying the better prognosis reported for endometrial cancer patients with coexistent adenomyosis.

In a recent systematic review and meta-analysis assessing 5573 endometrial cancer patients, adenomyosis has shown to be a common finding at histological examination of hysterectomy specimens, with a pooled prevalence of 22.6% (95% Confidence Interval (CI) 12.7–37.1%) [12]. Given such a high prevalence in women with endometrial cancer, several cohort studies have investigated the impact of adenomyosis on prognosis. A meta-analysis by An et al. including fourteen retrospective observational studies concluded that endometrial cancer patients with adenomyosis showed a significant increase in overall survival rate, indicating that coexistent adenomyosis might be associated with favorable endometrial cancer characteristics and considered a potential protective factor for the prognosis of endometrial cancer [21]. These results were confirmed in our recent meta-analysis which analyzed a population of 2505 women with endometrial cancer (553 with and 1952 without adenomyosis) and showed that endometrial cancer patients with adenomyosis had a halved risk of death and recurrence compared to endometrial cancer patients without adenomyosis [14]. In particular, compared to endometrial cancer patients without adenomyosis, endometrial cancer patients with coexistent adenomyosis showed a pooled hazard ratio (HR) of 0.533 (CI 95%, 0.329–0.864) for overall survival (OS) at univariate analysis, 0.536 (CI 95%, 0.334–0.859) for disease-free survival (DFS) at univariate analysis, and 0.875 (CI 95%, 0.331–2.315) for DFS at multivariate analysis [14].

The reported better prognosis of endometrial cancer with coexistent adenomyosis has been explained by different hypotheses. For example, adenomyosis has been linked to a specific profile of cytokines that includes higher levels of anti-tumoral cytokines and lower levels of oncogenic cytokines and growth factors; the local microenvironment could be impacted by this profile and tumor progression and invasiveness could be limited [21,22,23]. Moreover, the thickened endometrial stroma observed in the adenomyotic uterus following repeated inflammations might result in a block of endometrial cancer invasion in the myometrium [21,22,23]. Yet, a better prognosis might be subsequent to an early endometrial cancer diagnosis provided by adenomyosis-related symptoms (e.g., dysmenorrhea and abnormal uterine bleeding) [14]. However, these mechanisms have never been proved in large clinical studies. Furthermore, although the impact of adenomyosis on the risk of death from any cause in endometrial cancer patients was not evaluable at multivariate analysis in our recent meta-analysis [14], the risk of endometrial cancer recurrence in women with coexistent adenomyosis did not decrease significantly at multivariate analysis, suggesting that this association might be not independent. In other words, the association found at univariate analysis might be related to other prognostic factors that could be related to the coexistence of adenomyosis in endometrial cancer patients. Hence, it is necessary to investigate possible differences in clinical, histological and molecular prognostic factors between endometrial women with and without coexistent adenomyosis [14].

While comparisons of molecular prognostic factors between endometrial cancer patients with or without associated adenomyosis were not suitable when conducting a systematic review and meta-analysis of the literature because of the lack of individual eligible studies, differences in clinical and histological prognostic factors were evaluable by analyzing previous studies.

In particular, for clinical factors, in another systematic review and meta-analysis, we included eight studies with 5681 patients in the qualitative analysis, and seven studies with 4366 patients in the quantitative analysis. Specifically, the pooled mean difference in mean ± SD between endometrial cancer patients with and without adenomyosis was −1.19 (95% CI: −3.18 to 0.80; *p* = 0.24) for age, and 0.23 (95% CI: −0.62 to 1.07; *p* = 0.60) for BMI. When compared to endometrial cancer patients without adenomyosis, endometrial cancer patients with adenomyosis showed a pooled odds ratio (OR) of 1.53 (95% CI: 0.92 to 2.54; *p* = 0.10) for premenopausal status, and of 0.60 (95% CI: 0.41 to 0.87; *p* = 0.007) for nulliparity [8]. In conclusion, we found that there are no significant differences in clinical characteristics between endometrial cancer patients with and without adenomyosis in terms of age, BMI and premenopausal status, with the exception of nulliparity [8]. Therefore, clinical features seem not to underlie the better endometrial cancer prognosis of patients with adenomyosis compared to patients without adenomyosis.

On the other hand, in another systematic review and meta-analysis investigating the possible impact of histological prognostic factors on the better endometrial cancer prognosis in women with coexistent adenomyosis, we found that the prevalence of unfavorable histological prognostic factors significantly differed between endometrial cancer patients with and without coexistent adenomyosis [7]. In particular, a significantly decreased risk for FIGO grade 3, FIGO stages II-IV, LVSI and deep myometrial infiltration was found in endometrial cancer patients with adenomyosis compared to endometrial cancer patients without adenomyosis. In particular, when compared to endometrial cancer without adenomyosis, endometrial cancer with a adenomyosis showed a pooled relative risk of 0.77 (95% CI: 0.59, −1.00; *p* = 0.05) for the non-endometrioid histotype; 0.55 (95% CI: 0.42−0.71; *p* = 0.000001) for FIGO grade 3; 0.60 (95% CI: 0.42–0.85; *p* = 0.005) for FIGO stage II–IV; 0.75 (95% CI: 0.62–0.92; *p* = 0.004) for LVSI; and 0.65 (95% CI: 0.51–0.84; *p* = 0.001) for deep myometrial invasion [7].

Since pooled comparisons in the prevalence of the different molecular signatures between endometrial cancer patients with and without coexistent adenomyosis were not suitable due to the lack of previous studies on the topic in the literature, we carried out the present study with this aim. In particular, we assessed our study population and we found no significant difference in the prevalence of molecular groups at poor and intermediate prognosis between the adenomyosis and no adenomyosis groups of endometrial cancer patients.

Thus, conventional histological prognostic factors rather than clinical and molecular ones might explain the better survival outcomes of endometrial cancer patients with coexisting adenomyosis. Such a finding seems to be in line with the results of recent studies demonstrating that conventional histological prognostic factors retain a prognostic value independent of the molecular signature. In fact, although TCGA/ProMisE molecular groups have shown the potential to reduce past under- and overtreatment of EC patients [18,24], some studies reported that histotype, deep myometrial invasion and LVSI retain a prognostic value independent of the molecular signature [1,25,26]. In particular, non-endometrioid EC has shown a worse prognosis in each TCGA/ProMisE group [25], deep myometrial invasion has been shown to independently affect the risk of recurrence [1], and LVSI has been shown to independently increase the risk of death from any cause, death due to EC and recurrent or progressive disease by 1.5–2 times [26].

The integration of conventional histopathological and novel molecular features in the risk stratification of endometrial cancer women appears to be supported by these findings, as recommended by the 2020 ESGO/ESTRO/ESP guidelines and the 2023 FIGO staging of endometrial cancer [27,28].

### 4.2. Strengths and Limitations of This Study and Future Perspectives

To the best of our knowledge, this study may be the first to investigate the impact of the molecular signature on the prognosis of endometrial cancer patients with coexistent adenomyosis.

However, despite their novelty, some limitations might impact our findings. Indeed, the retrospective design and the relatively small sample size are some of the drawbacks of this study. Yet, a major limitation might consist in the lack of information about the prevalence of the NSMP and POLE-mt signatures in the study population. In fact, our centers restrict POLE testing to only those patients in whom this would alter adjuvant therapy recommendations in order to restrict sequencing costs. In other words, as the POLE signature was not available for all endometrial cancer patients in our centers, a retrospective calculation of the prevalence of the POLE signature (and subsequently of the NSMP signature) was not feasible. In order to assess the prevalence of the NSMP and POLE-mt signatures, future prospective studies assessing mutations in the exonuclease domain of Polymerase-ε on all endometrial cancers samples are necessary.

In the near future, we hope that an increasingly tailored risk assessment based on an integration of clinical, histological and molecular prognostic factors will direct the preoperative, surgical and postoperative management of endometrial cancer patients.

### 4.3. Conclusions

The prevalence of p53-abn and MMR-d signatures does not differ between endometrial cancer patients with and without coexistent adenomyosis. Therefore, the molecular signature might not explain the better prognosis associated with coexistent adenomyosis in endometrial cancer patients.

In order to confirm these findings and further investigate the topic, larger prospective studies, also performing gene sequencing for the detection of POLE mutations on all samples, are necessary.

## Figures and Tables

**Table 1:** Characteristics of the study population.

Characteristic	All (n = 147)	Adenomyosis		*p*-Value
		Yes (n = 38)	No (n = 109)	
Age, years	64 (28–92)	62 (38–84)	64 (28–92)	0.6729
BMI				0.6303
<25 kg/m^2^	41 (27.9)	8 (21.1)	33 (30.2)	
25–30 kg/m^2^	46 (31.3)	14 (36.8)	32 (29.4)	
30–35 kg/m^2^	30 (20.4)	7 (18.4)	23 (21.1)	
>35 kg/m^2^	30 (20.4)	9 (23.7)	21 (19.3)	
FIGO stage				0.4384
I–II	124 (84.3)	34 (89.5)	90 (82.6)	
III–IV	23 (15.6)	4 (10.5)	19 (17.4)	
Histotype				0.5931
Endometrioid	126 (85.7)	34 (89.5)	92 (84.4)	
Not endometrioid	21 (14.3)	4 (10.5)	17 (15.6)	
Tumor grade				1.0000
G1–G2	116 (78.9)	30 (78.9)	86 (78.9)	
G3	31 (21.1)	8 (21.1)	23 (21.1)	
Lymphovascular Invasion				0.5598
Negative	87 (59.2)	25 (65.8)	62 (56.9)	
Focal	36 (24.5)	7 (18.4)	29 (26.6)	
Diffuse	24 (16.3)	6 (15.8)	18 (16.5)	
Sentinel Lymph Node				0.9326
Negative	123 (83.7)	32 (84.2)	91 (83.5)	
Positive	14 (9.5)	4 (10.5)	10 (9.2)	
Missing	10 (6.8)	2 (5.3)	8 (7.3)	
Pelvic Lymphadenectomy				0.4185
Yes	32 (21.8)	6 (15.8)	26 (23.8)	
No	115 (78.2)	32 (84.2)	83 (76.2)	
Paraortic Lymphadenectomy				0.6984
Yes	15 (10.2)	5 (13.2)	10 (9.2)	
No	132 (89.8)	33 (86.8)	99 (90.8)	
Adjuvant Therapy				0.0505
Yes	67 (45.6)	23 (60.5)	44 (40.4)	
No	80 (54.4)	15 (39.5)	65 (59.6)	
Recurrence	5 (3.4)	2 (5.2)	3 (2.7)	-
Death from any cause	0	0	0	-
Death due to tumor	1 (0.6)	1 (2.6)	0	-

Values are given as numbers (% or range) unless otherwise noted. BMI: Body Mass Index; FIGO: International Federation of Gynecology and Obstetrics.

**Table 2:** Molecular signatures at poor and intermediate prognosis in the study population.

Molecular Signature	All (n = 147)	Adenomyosis		*p*-Value
		Yes (n = 38)	No (n = 109)	
MMR-d	37 (25.2)	12 (31.6)	25 (22.9)	0.2880
p53-abn	12 (8.2)	3 (7.9)	9 (8.3)	1.0000

Values are given as numbers (% or range) unless otherwise noted. MMR-d: mismatch repair deficient expression; p53-abn: p53 abnormal expression.

## Data Availability

The data that support the findings of this study are available from the corresponding authors (A.R. and R.P.) upon reasonable request.
